# Spiritual needs and quality of life of cancer patients

**DOI:** 10.1007/s00520-026-11012-2

**Published:** 2026-07-23

**Authors:** Diego de Araujo Toloi, Rachel P. Riechelmann

**Affiliations:** 1 Post Graduate Program in Oncology, A.C. Camargo Cancer Center, São Paulo, Brazil; 2 Department of Clinical Oncology, A.C. Camargo Cancer Center, São Paulo, Brazil

**Keywords:** Spirituality, Religiosity, Quality of life, Cancer

## Abstract

**Purpose:**

Spirituality is important in cancer care. Few studies have assessed the spiritual needs (SN) of cancer patients. The aim of this study was to assess the SN of cancer patients and evaluate its relationship with their health-related quality of life (HRQOL).

**Methods:**

We conducted a unicenter cross-sectional study of patients undergoing cancer treatment. We used validated instruments (Duke University Religion Index—DUREL, Functional Assessment of Chronic Illness Therapy Spiritual Well Being—FACIT-SP, and Spiritual Needs Assessment for Patients—SNAP) and a questionnaire about the healthcare team’s approach to spirituality.

**Results:**

From March to October/2022, 150 patients were included: 75 receiving curative-intent and 75 under palliative cancer-directed therapy. Patients who wished a spirituality approach by the healthcare team had higher SN than patients who did not want it (*p* < 0.0001), but lower HRQOL scores (*p* = 0.004). Neither SN nor HRQOL differed according to treatment intention (*p* = 0.444 and *p* = 0.159 respectively). There was a weak correlation between SN and HRQOL. The answers about the wish for a spirituality approach were grouped in four categories: (1) approach by the health team, (2) comprehension of spirituality/religiosity, (3) impact of the spirituality/religiosity approach, and (4) self-referential or other-referential responses. Participants perceived very few discussions on spirituality.

**Conclusion:**

In our study, patients who wished a spiritual approach had higher SN and lower QOL. SN were not related to cancer treatment intentions. Further studies could evaluate screening and interventions in this area.

**Supplementary Information:**

The online version contains supplementary material available at 10.1007/s00520-026-11012-2.

## Introduction

Spirituality is a dynamic aspect of human life related to the search for meaning, purpose, and transcendence. It involves connecting with the present moment, oneself, others, nature, and the sacred. Although religion can be a tool for spirituality, it is not a requirement or synonym. Therefore, the concept of spirituality should be inclusive, embracing religious and non-religious aspects of individuals [1–3].

In healthcare, spirituality is associated with patients’ quality of life, coping, and the decision-making. The World Health Organization (WHO) recognizes spirituality as part of comprehensive care in its definitions of quality of life and palliative care. The WHO defines quality of life as including the following domains: physical health, psychological well-being, level of independence, social relationships, environment, and spirituality, religion and personal beliefs. However, there is no widely accepted definition of spirituality that is both inclusive and useful for research and clinical practice [4–9].

Recently, the importance of spirituality in health research has increased. Researchers have developed instruments that focus on cognitive, behavioral, and affective dimensions. Studies have covered topics ranging from spiritual well-being to coping with illness. The concept of spiritual needs is complex. The Spiritual Needs Assessment for Patients (SNAP) was developed to assess unmet spiritual needs through psychosocial, religious, and spiritual dimensions. It has been validated in Chinese, Brazilian Portuguese, and Sinhalese. Identifying instruments to properly assess spiritual needs is recognized as a research priority [6, 10–16].

Spirituality is an important aspect of cancer patient care. The natural history of the disease is associated with uncertainty and suffering. Research indicates that spirituality influences quality of life and spiritual well-being, coping mechanisms, and clinical decision-making [17–22].

Published by the Brazilian Society of Clinical Oncology (SBOC), the 2022 “Spirituality in Oncology” consensus presents strategies and tools for providing spiritual care to cancer patients. Nevertheless, the consensus emphasizes the lack of research of oncology and spirituality, and stresses the importance of studying the spiritual needs of cancer patients [23].

Spiritual needs differ conceptually from spiritual distress. While spiritual distress involves suffering related to meaning, purpose, or connection, spiritual needs encompass broader psychosocial, religious, and spiritual desires that may exist without distress [12, 23].

Although spirituality, quality of life, and religiosity may be associated, few studies have examined their relationship with spiritual needs [4, 6, 9, 19]. Likewise, despite evidence linking sociodemographic and clinical characteristics to spirituality, data on their association with spiritual needs, including patients’ desire for spiritual discussions, remain limited [18, 23].

We designed this study to characterize the spiritual needs of cancer patients undergoing treatment, using the validated Brazilian Portuguese version of the SNAP questionnaire. The secondary objectives were to describe the religious and spiritual profiles of these patients, evaluate the relationship between their spiritual needs and quality of life, and assess the influence of religion on these needs and quality of life.

## Methods

### Design

We conducted a monocentric descriptive and analytical cross-sectional study with cancer patients. We integrated quantitative and qualitative data through complementarity using a mixed-methods approach.

### Participants and setting

We enrolled outpatients undergoing oncological treatment with curative or palliative intent at the A.C.Camargo Cancer Center, São Paulo, Brazil. The recruitment period was from March to October 2022.

Eligible participants were adults aged 18 years or over with a diagnosis of a solid tumor and at least 4 years of education. We excluded patients whose performance status would compromise their ability to complete the questionnaires, and patients who were disoriented or confused.

We estimated the sample size using the systematic review by Bai et al. [19]. This review included studies that assessed spirituality in oncology using questionnaires, with an estimated average sample size of 96.53. We determined the final sample size to 150 participants based on convenience and feasibility. Participants were selected using consecutive non-probability sampling. This sample size ensured a robust characterization of the spiritual needs of cancer outpatients within the Brazilian context.

This study was approved by the Research Ethics Committee at Fundação Antônio Prudente – A.C.Camargo Cancer Center (2853/20, June 2020). All included patients signed an Informed Consent Form.

### Data collection

We used a brief questionnaire on the healthcare team’s approach to spirituality and sociodemographic data, the Duke University Religion Index (DUREL), the Functional Assessment of Chronic Illness Therapy Spiritual Well Being (FACIT-SP), and the SNAP. The DUREL, FACIT-SP, and SNAP versions used were all self-completion questionnaires and validated for Brazilian Portuguese. Permission has been obtained for use of the questionnaires.

### Instruments

#### Questionnaire on the healthcare team’s approach to spirituality and sociodemographic data and clinical characteristics

The questionnaire assessed the following demographic characteristics: age, gender, self-reported race, marital status, and level of education. There were four closed-ended questions: whether the patient had a religion, religious affiliation(s), how often the healthcare team had conversations about spirituality or religiosity during follow-up, and whether the patient desired these conversations (“Would you like your team to talk about spirituality or religiosity?”). After this last question, there was an open-ended question asking the patient to justify their answer.

Clinical data were collected from electronic records, including primary tumor, staging, and Eastern Cooperative Oncology Group (ECOG) performance status. The objective of the oncological treatment (curative or palliative) was extracted directly from electronic medical records and was based on the formal clinical evaluations conducted by the patients’ medical oncologists.

#### DUREL

This index assesses the participant’s religiosity in three dimensions: organizational religiosity (OR), non-organizational religiosity (NOR), and intrinsic religiosity (IR) [24]. OR and NOR can range from 1 to 6, while IR can range from 3 to 15 (the higher the index the greater the religiosity).

#### FACIT-SP

The FACIT-SP comprises 39 items, each of which is answered using a five-point Likert scale. The score can range from 0 to 156 (the higher the result, the better the quality of life).

#### SNAP

The SNAP questionnaire evaluates current or possible future spiritual needs based on statements in three domains: psychosocial, spiritual, and religious. The total score can range from 23 to 92 (the higher the total score, the greater the spiritual need) [16, 25].

All instruments were self-completed and the total time for completion was estimated as 30 min. Patients completed the questionnaires while they were receiving intravenous cancer-directed therapy at the Chemotherapy Unit.

### Statistical analysis

We performed a descriptive statistical analysis of the SNAP data, presenting the total sample scores by intended treatment. The normality test and the t-test for independent samples were used to compare the spiritual needs of the patient groups. Inferential statistical analysis was used to evaluate factors related to spiritual needs, including age, level of education, self-declared gender, marital status, and the desire for an approach to spirituality/religiosity.

We conducted a descriptive statistical analysis of the questionnaire regarding the healthcare team’s approach to spirituality and the DUREL data. Inferential statistical analysis was used to compare religiosity rates between patient groups and according to the wish to address spirituality/religiosity.

We performed a descriptive statistical analysis of the FACIT-SP data. Inferential statistical analysis was used to compare quality of life scores of patient groups and according to the wish to address spirituality/religiosity.

We used Pearson’s correlation coefficient to analyze the relationships between spiritual needs and quality of life, spiritual needs and religiosity, and religiosity and quality of life.

The results were statistically significant if the *p*-value was less than 0.05.

### Analytic approach

The phenomenon under investigation was the patient’s justification for their wish for a spiritual approach by the healthcare team. We analyzed the answers to justify the wish for an approach from the healthcare team using Content Analysis (CA) [26]. In accordance with Bardin’s Content Analysis (CA), the pre-analysis included the corpus document selection, the preparation of material and the floating reading of the material. The material exploration phase consisted of the coding and categorization process.

### Missing data

We checked for missing responses after completing the questionnaire and informed the participant. If there was still no response, this was documented in the questionnaire itself. With regard to missing data in the P-DUREL for OR and NOR, the presence of missing data would make it impossible to conduct analyses that require these dimensions, respectively. For the three-item IR dimension, Person Mean (PM) imputation was applied when one item was missing, using the mean of the remaining two items. Participants with two or more missing IR items were excluded from analyses involving this dimension. The FACIT-SP score for each subscale was calculated in accordance with the instructions for the instrument. Missing SNAP data was imputed using the subscale mean; if half or more items were missing, the data were excluded from the analysis.

## Results

A total of 179 patients were screened during the study period. Reasons for refusal included physical factors (e.g., the need for glasses), factors relating to the research itself (e.g., the topic itself), and availability issues (e.g., lack of time). The final sample size was 150 participants undergoing active oncological treatment (75 of curative intent and 75 of palliative intent).

The mean age of the participants was 55.76 years. The majority was female (58%), self-identified as White (72%), and were married (70.7%). The curative intent group predominantly comprised patients with breast cancer (50.67%), while the palliative intent group was mostly represented by patients with gastrointestinal tract cancers (42.67%). The sample predominantly comprised of patients with ECOG performance status 0 or 1 (90%). Table 1 describes the characteristics of the study participants.

**Table 1:** Participants’ socio demographic, clinical, and religious and spiritual characteristics

Variable		Total	Curative	Palliative	*p*
Age (years)	Mean	55.76	53.19	58.33	
	Median	56	53	53	0.016
	Min-max	19-84	19-84	33-80	
Gender *n* (%)	Female	87 (58%)	51 (68%)	36(48%)	0.021
	Male	63 (42%)	24 (32%)	39 (52%)	
Self-reported race *n* (%)	White	108 (72%)	57 (76%)	51 (68%)	0.674
	Brown	19 (12,67%)	9 (12%)	10 (13.34%)	
	Black	12 (8%)	5 (6.67%)	7 (9.33%)	
	Yellow	11 (7.33%)	4 (5.33%)	7 (9.33%)	
Marital status *N* (%)	Married	106 (70,7%)	54 (72%)	52 (69.33%)	
	Not-married:				
	Separated	22 (14,67%)	10 (13.33%)	12 (16%)	0.858
	Single	15 (10%)	10 (13.33%)	5 (6.67%)	
	Widowed	7 (4.66%)	1 (1.34%)	6 (8%)	
Education (full years)	Mean	14.31	15.1	13.52	
	Median	15	15.5	15	0.027
	Min-max	4-25	4-25	4-22	
	Low:				
	1-5 years n (%)	8 (5.33%)	2 (2.67%)	6 (8%)	
	6-9 years n (%)	11 (7.33%)	6 (8%)	5 (6.67%)	
	10-12 years n(%)	31 (20,67%)	14 (18.67%)	17 (22.67%)	0.376
	High:				
	≥13 years n (%)	100(66.7%)	53 (70.66%)	47 (62.66%)	
Primary cancer site *n* (%)	Breast	53 (35,33%)	38 (50.67%)	15 (20%)	
	Gastrointestinal	46 (30,67%)	14 (18.67%)	32 (42.67%)	
	Skin	15 (10%)	4 (5.33%)	11 (14.67%)	
	Head and neck	10 (6.67%)	9 (12%)	1 (1.34%)	
	Gynecological	9 (6%)	3 (4%)	6 (8%)	
	Lung	8 (5.33%)	3 (4%)	5 (6.67%)	
	Genitourinary	5 (3.33%)	3 (4%)	2 (2.67%)	
	Sarcoma	3 (2%)	1 (1.34%)	2 (2.67%)	
	Thyroid	1 (0.67%)		1 (1.34%)	
Staging *n* (%)	I	9 (6%)	9 (12%)		
	II	19 (12,67%)	19 (25.33%)		
	III	45 (30%)	45 (60%)		
	IV	77 (51,33%)	2 (2.67%)	75 (100%)	
ECOG *n* (%)	0	90 (60%)	55 (73.33%)	35 (46.67%)	
	1	45 (30%)	16 (21.33%)	29 (38.67%)	0.004
	2	12(8%)	4 (5.34%)	8 (10.66%)	
	3	3 (2%)		3 (4%)	
Religion	No	10 (6.6%)	3 (4%)	7 (9.3%)	0.326
	Yes	140(93%)	72 (96%)	68(90.6%)	
Religious affiliation	Catholicism	60 (40%)	30 (40%)	30 (40%)	
	Spiritism	22(14.6%)	11 (14.6%)	11(14.6%)	
	Protestantism	18 (12%)	9 (12%)	9 (12%)	
	Judaism	1 (0.6%)	1 (1.3%)		
	Buddhism	1 (0.6%)		1 (1.3%)	
	Believ in God	6 (4%)	2 (2.6%)	4 (5.3%)	
	Atheism	1 (0.6%)		1 (1.3%)	
	Other	13 (8.6%)	7 (9.3%)	6 (8%)	
	Catholicism and Spiritism	20(13.3%)	11 (14.6%)	9 (12%)	
	Believe in God and Other	2 (1.3%)		2 (2.6%)	
	Catholicism, Protestantism	1 (0.6%)	1 (1.3%)		
	Catholicism, Buddhism	1 (0.6%)		1 (1.3%)	
	Catholicism, Believ in God	1 (0.6%)	1 (1.3%)		
	Spiritism, Believ in God	1 (0.6%)	1 (1.3%)		
	Atheism, Other	1 (0.6%)		1 (1.3%)	
	Spiritism, Buddhism, Other	1 (0.6%)	1 (1.3%)		
Does your healthcare team discuss spirituality or religiosity?	No	97(64.6%)	47(62.6%)	50(66.6%)	
	Rarely	30 (20%)	15 (20%)	15 (20%)	
	Sometimes	18 (12%)	11(14.6%)	7 (9.3%)	0.852
	Often	4 (2.6%)	2 (2.6%)	2 (2.6%)	
	No, Often	1 (0.6%)		1 (1.3%)	
Would you like the healthcare team to talk?	Yes	82(54.6%)	35(46.6%)	47(62.6%)	0.099
	No	64(42.6%)	37(49.3%)	27 (36%)	
	Not answer	3 (2%)	2 (2.6%)	1 (1.3%)	
	Yes and No	1 (0.66%)	1 (1.3%)		

*ECOG* Eastern Cooperative Oncology Group performance status scale

The missing data are shown in Supplemental Table 1.

### Spiritual needs of cancer patients

Table 2 shows the results of the SNAP questionnaire administration. The spiritual needs domain and total score are presented in the first column. There was no statistical difference according to treatment intention. Spiritual needs did not differ according to age (*r* = 0.059), education level (*p* = 0.999), or marital status (*p* = 0.528). Females had higher scores for spiritual needs in the psychosocial domain compared to males (*p* = 0.042)—Supplemental Table 2. Patients who answered “yes” to the question, “Would you like your team to talk about spirituality or religiosity?”, had higher total spiritual needs, psychosocial, spiritual, and religious needs scores than patients who answered “no” (*p* < 0.0001) (Table 2).

**Table 2:** Spiritual needs according to treatment intention and patient’s wishes regarding the healthcare team approach (mean and min–max)

		Treatment intention			Would you like the healthcare team to talk?		
	Total	Curative (*n*=75)	Palliative (*n*=75)	*p*	No (*n*=64)	Yes (*n*=82)	*p*
Psychosocialneeds	14.25(5-20)	14.26(5-20)	14.23(7-20)	0.951	13.03(5-20)	15.29(9-20)	<0.0001
Spiritualneeds	34.72(13-52)	34.37(13-52)	35.08(13-51)	0.619	30.84(13-46)	37.74(18-52)	<0.0001
Religiousneeds	12.13(5-20)	11.57(5-20)	12.69(5-20)	0.112	10.06(5-20)	13.64(5-20)	<0.0001
SNAP score	61.09(23-92)	60.21(23-92)	61.96(25-88)	0.444	53.89(23-81)	66.69(34-92)	<0.0001

*SNAP* Spiritual Needs Assessment for Patients

### The religious and spiritual profiles of cancer patients

The majority of participants had a religion, primarily Catholicism or Spiritism. Twenty-eight participants (18.67%) indicated more than one religious affiliation. Most participants responded that spirituality or religiosity is rarely or never discussed by the health care team. The palliative care group had a greater wish for this approach than the curative care group: 62.66% and 46.66% respectively (*p* = 0.099). These data are presented in Table 1. There was no statistically significant difference regarding the wish for address spirituality/religiosity according to the participant’s gender (*p* > 0.999).

The DUREL religiosity indexes showed similar values between the groups of participants. The OR and NOR indices were higher for participants who wished a spirituality approach (*p* = 0.046 and *p* = 0.006, respectively) (Table 3).

**Table 3:** The DUREL religiosity indexes according to the treatment intention and patient’s wishes regarding the healthcare team approach (mean and min–max)

		Treatment intention			Would you like the healthcare team to talk?		
	Total	Curative (*n*=75)	Palliative (*n*=75)	*p*	No (*n*=64)	Yes (*n*=82)	*p*
OR	3.61(1-6)	3.68(1-6)	3.54(1-6)	0.549	3.34(1-6)	3.81(1-6)	0.046
NOR	4.13(1-6)	4.25(1-6)	4.01(1-6)	0.339	3.73(1-6)	4.45(1-6)	0.006
IR	12.85(3-15)	12.96(6-15)	12.73(3-15)	0.580	12.61(3-15)	13.06(3-15)	0.284

*OR* organizational religiosity, *NOR* non-organizational religiosity, *IR* intrinsic religiosity

### Responses from cancer patients regarding the healthcare team’s approach to spirituality

A content analysis was performed to qualitatively analyze patients’ responses regarding their wish to address spirituality.

In the pre-analysis we included the 123 answers in the corpus document selections, performed the preparation of material and the floating reading.

**Fig. 1:**
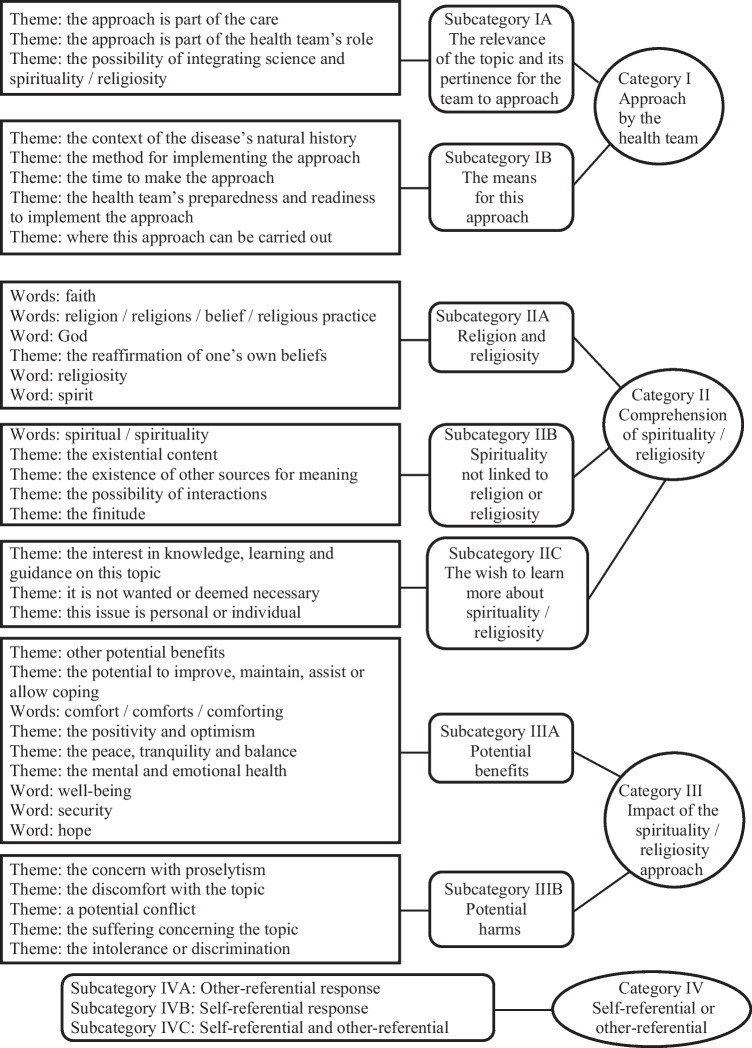
Coding, subcategorization, and categorization

In the material exploration phase, we transformed raw data (the 123 answers) into meaningful 39 units of analyses, comprising: 26 themes, 10 word groups and 3 references from response, which classify the subject references employed by participants in their writing. We defined ten subcategories and organized four categories: (1) approach by the health team, (2) comprehension of spirituality/religiosity, (3) impact of the spirituality/religiosity approach, and (4) self-referential or other-referential responses. Figure 1 illustrates this organization, and Supplemental Table 3 provides illustrative quotes. The treatment of the results was also processed (Supplemental Table 4).

### The relationship between spiritual needs and quality of life

There was no difference regarding quality of life between the groups of participants (*p* = 0.159) (Table 4). There was a statistically significant difference regarding quality of life according to the wish for a spirituality approach between the FACIT-G score (*p* = 0.001) and the FACIT-SP score (*p* = 0.004) indicating a lower quality of life for participants who desired an approach of spirituality (Table 4). Pearson’s correlation coefficients between participants’ spiritual needs and their quality of life scores indicated weak (less than 0.4) and mostly negative correlations (Table 5).

**Table 4:** FACIT-SP scores according to the patient’s wishes regarding the healthcare team approach (mean and min–max)

		Treatment intention			Would you like the healthcare team to talk?		
	Total	Curative (*n*=75)	Palliative (*n*=75)	*p*	No (*n*=64)	Yes (*n*=82)	*p*
Physical	19.69(1-28)	20.36(9-28)	19.03(1-28)	0.174	21.40(1-28)	18.34(1-28)	0.002
Social and Family	22.25(5-28)	22.79(9-28)	21.71(5-28)	0.132	23.21(15-28)	21.71(5-28)	0.037
Emotional	18.32(7-24)	18.81(9-24)	17.83(7-24)	0.149	19.08(8-24)	17.69(7-24)	0.045
Functional	18.74(1-28)	18.73(6-28)	18.75(1-28)	0.982	19.99(7-28)	17.92(1-28)	0.018
FACIT-G	78.99(35-106)	80.69(45-105)	77.31(35-106)	0.17	83.69(48-106)	75.66(35-102)	0.001
Meaning and peace	25.24(8-32)	25.48(8-32)	25(13-32)	0.57	26.57(16-32)	24.37(8-32)	0.010
Faith	12.79(2-16)	13.22(4-16)	12.36(2-16)	0.116	12.53(2-16)	13.03(5-16)	0.382
Spiritual	38.04(18-48)	38.7(19-48)	37.38(18-48)	0.258	39.1(21-48)	37.42(18-48)	0.159
FACIT-SP	117.04(56-154)	119.39(64-150)	114.69(56-154)	0.159	122.79(78-154)	113.07(56-150)	0.004

**Table 5:** Pearson correlation coefficients among spiritual needs, quality of life and religiosity indexes

	Psychosocial needs	Spiritual needs	Religious needs	SNAP score
Physical	-0.290	-0.247	-0.142	-0.258
Social and Family	-0.178	-0.228	-0.103	-0.211
Emotional	-0.306	-0.416	-0.239	-0.396
Functional	-0.236	-0.305	-0.210	-0.302
FACIT-G	-0.335	-0.387	-0.226	-0.380
Meaning and peace	-0.232	-0.315	-0.113	-0.279
Faith	-0.089	-0.131	0.224	0.170
Spiritual	-0.126	-0.166	0.022	-0.123
FACIT-SP	-0.291	-0.344	-0.159	-0.323
OR	0.077	0.184	0.361	0.244
NOR	0.083	0.164	0.288	0.208
IR	0.178	0.257	0.267	0.280

*OR* organizational religiosity, *NOR* non-organizational religiosity, *IR* intrinsic religiosity

### The relationship between religiosity, spiritual needs, and quality of life

Pearson’s correlation coefficients between participants’ DUREL religiosity indexes and their spiritual needs scores indicated weak (less than 0.4) and negative correlations (Table 5). Supplemental Table 5 shows the correlation between participants’ DUREL religiosity indexes and their quality of life scores. The spiritual well-being faith showed moderate and positive correlations (between 0.4 and 0.7) with the three religiosity indices.

## Discussion

Our study describes the spiritual needs of cancer patients and their association with quality of life. Patients who wished to discuss spirituality or religiosity with the healthcare team had higher spiritual needs and lower quality of life, regardless of the treatment intention. At the same time, while nearly half of participants wished their health care team to address spirituality, their perceptions were that some discussions on spirituality occurred in only 20% of cases and did not occur at all in almost two thirds of cases.

When considering studies of the spiritual needs of cancer patients, the discrepancy between the data from the Portuguese validation study and that from previous SNAP studies (the questionnaire development study and the validation studies for Chinese and Sinhalese patients) could suggest that Brazilian cancer patients have greater spiritual needs. However, the present study had a larger sample size and was therefore more representative of the outpatient cancer patient population, producing lower mean scores than the Portuguese validation study. The current study’s findings were closer to those obtained in the questionnaire development study and the Chinese and Sinhalese validation studies [16, 25, 27, 28].

The results of our study showed no difference in spiritual needs between the groups of participants undergoing curative or palliative treatment. The literature addressing direct comparisons of spiritual needs according to cancer treatment intent using validated instruments remains scarce. Another study of hospitalized patients used the SNAP questionnaire and assessed the indication for palliative care using the World Health Organization Palliative Needs Tool (NECPAL). Patients without an indication for palliative care had higher spiritual needs scores. The authors discussed the hypothesis that participants with an indication for palliative care have more frequent discussions about their health condition, then it might result in fewer spiritual needs during hospitalization [29].

In this sense, the lack of differences in spiritual needs according to cancer treatment intent highlights the need to improve health care understanding. This requires us to broaden our biological focus and consider patients’ biographies. Moreover, we need to pay attention to how these aspects change throughout treatment and care processes. This may also explain why the socio-demographic variables were not significant for spiritual needs.

The evaluation of the gender variable revealed that females had higher spiritual needs scores than males. Religious practices and traditions offer different representations of socially expected gender roles on these issues [30]. Therefore, these influences should be considered when analyzing data obtained in multicultural contexts. Studies on spirituality within these social and cultural contexts must also consider the diversity of gender identities, thus fostering a comprehensive and diverse approach to spiritual access and care.

Our analyses revealed no differences in willingness to discuss spirituality/religiosity with the team based on curative or palliative intent or participant gender. This finding supports that spiritual care should not be based solely on the natural history of the disease or biological factors; it should also consider patients’ biographical aspects.

In our study 54.6% of participants wanted the team to address spirituality/religiosity. However, the team rarely had this conversation for 20% of patients and they did not have it for 64.6%. According to a narrative review published in 2023, cancer patients desire assistance from their healthcare team in addressing their spiritual needs. However, this rarely happens [31].

Additionally, the association between patients’ wish for an approach from the healthcare team and their spiritual needs scores highlights the need for further research about spiritual care. The themes that emerged from our qualitative analysis reinforce this research demand.

There is a lack of gold-standard instruments for the psychometric evaluation of spiritual needs questionnaires, as well as validated and culture-adapted tools [32]. Noteworthy, ethical considerations must be considered when using these instruments and that appropriate guidance must be provided to healthcare team members regarding this approach [33].

The 2021 ESMO guideline affirms the importance of identifying spiritual distress throughout the disease trajectory [34]. Similarly, the NCCN guideline recommends incorporating the spiritual dimension into distress screening and emphasizes the importance of validating spiritual screening instruments [35]. The 2017 ASCO guideline also recommends that teams explore how patients’ spirituality affects their decision-making [36].

In line with these recommendations, this study presents a potential screening question for cancer patients, “Would you like your team to talk about spirituality or religiosity?” The “yes” or “no” answer correlates with the score on a validated, culturally adapted Brazilian questionnaire on spiritual needs. This finding aligns with existing recommendations and paves the way for future studies incorporating screening questions and subsequent use of SNAP.

Qualitative studies of cancer patients have identified the need for spiritual support and the importance of healthcare professionals’ attitudes toward spiritual care [37–39]. The present study also supports spirituality and religiosity potential in cancer patients’ trajectories. The “approach by the health team” category allows strategies to be considered in order to establish an approach. The “comprehension of spirituality/religiosity” category relates to a broad and inclusive concept of spirituality. The “impact of the spirituality/religiosity approach” category comprises benefits related to coping and well-being, as evidenced by the literature [18–20]. The themes related to harm emphasize the necessary attention for this approach, and proselytism is a particular concern. These results highlight the importance of careful consideration and preparation when providing spiritual care to cancer patients [23]. In the “self-referential or other-referential responses” category, references to the collective (other-referential) were more prevalent than personal references (self-referential) in responses from patients who wished this approach. This may be related to the collective understanding and potential social support offered by spirituality.

Assessing the quality of life of cancer patients using questionnaires is complex, and it may be related to changes in patients’ values or the evolving concept of quality of life throughout the course of the disease [40]. In our study, patients undergoing treatment with curative intent had higher quality of life scores, though the difference was not statistically significant compared to those undergoing treatment with palliative intent.

We observed lower quality of life scores among patients wishing to discuss spirituality/religiosity with the healthcare team, and these differences were statistically significant. One hypothesis is that spiritual needs influence patients’ perception of quality of life. Another hypothesis is that patients with a poorer quality of life seek spiritual resources to reevaluate their experiences. This search can be facilitated by the approach of the healthcare team. Future longitudinal studies could improve this understanding.

The DUREL questionnaire revealed high levels of intrinsic religiosity in both patient groups. Patients who wished to talk with the team about spirituality or religiosity had higher rates of organizational and non-organizational religiosity. These results provide information about the potential of religious resources for cancer patients. The role and quality of these practices in participants’ coping strategies should be examined further.

The DUREL questionnaire development study discussed how it is a complex construction, suggesting that it should be used alongside other measures [41]. The present study assesses both religiosity and spirituality using different measures and together with patients’ quality of life.

The distinction between different religiosity indexes may be more theoretical than practical, particularly considering the dynamic of cancer patients’ relationships with religiosity throughout the disease course. Thus, the positive and moderate correlation found in our study between the three religiosity indices and patients’ well-being faith is interesting.

Cancer patients may be affected by the impact of diagnosis and treatment on their beliefs. Thus, they may consider it important to discuss religiosity with various interlocutors, including the healthcare team [42]. However, our study revealed a weak positive correlation between religiosity and spiritual needs.

Our study is limited by its single-center design and lack of a longitudinal evaluation (especially because spiritual needs are dynamic). Since the study participants came from private healthcare providers, the data obtained may not be applicable to other healthcare contexts, particularly public health. In this regard, given the predominance of participants with an ECOG performance status of 0 or 1, any potential differences may require a more careful analysis to be adequately identified. Another limitation concerns the handling of missing data in questionnaire-based datasets. Additionally, patients with insufficient reading, comprehension, and writing skills were excluded. From a bioethical perspective, it is important to consider assistive technology and universal design to ensure that access is available for all patients in future studies.

It is worth highlighting that our study employed a combination of instruments, questionnaires, and quantitative and qualitative analyses. Mixed-methods studies have been recognized for investigating complex phenomena, making them suitable for contexts where literature is lacking [43–45]. In this sense, our results uniquely contribute to the topic of spirituality in cancer care by demonstrating the importance and feasibility of various methodologies in this area.

## Conclusion

Spirituality is an important aspect of cancer patient care. Our study found that patients who wished to discuss spirituality or religiosity with the healthcare team had greater spiritual needs and a lower quality of life, regardless of cancer treatment intent. Also, we observed that very few discussions on spirituality were perceived by participants. Future studies should evaluate screening and the impact of intervention strategies for spiritual needs in oncology care.

## Supplementary Information

Below is the link to the electronic supplementary material.

ESM 1 (DOCX 25.3 KB)

## Data Availability

The data from the present study are available from the corresponding author, but restrictions may apply to the availability of these data to ensure maintenance of participant anonymity.
