# Supplementary material for: Spiritual needs and quality of life of cancer patients
Source: Support Care Cancer. 2026 Jul 23;34(8):795. doi: 10.1007/s00520-026-11012-2 (PMC13396117; doi:10.1007/s00520-026-11012-2)
Supplement: Supplementary file 1 — (DOCX 25.3 KB) [file 520_2026_11012_MOESM1_ESM.docx]

**Supplemental Table 1** – Missing data.

| Instrument |  | Total | Curative | Palliative |
| --- | --- | --- | --- | --- |
| Would you like the healthcare team | Yes / No | 3 | 2 | 1 |
| to talk about spirituality / religiosity? | Why? | 27 | 14 | 13 |
| DUREL | OR | 1 |  | 1 |
|  | IR | 1 |  | 1 |
| FACIT-SP - GP | GP5 | 2 | 2 |  |
| FACIT-SP - GS | GS1 | 1 |  | 1 |
|  | GS2 | 1 |  | 1 |
|  | GS3 | 3 | 2 | 1 |
|  | GS4 | 3 |  | 3 |
|  | GS5 | 1 | 1 |  |
|  | GS6 | 9 | 3 | 6 |
|  | GS7 | 8 | 4 | 4 |
| FACIT-SP - GE | GE3 | 3 | 2 | 1 |
|  | GE4 | 3 | 1 | 2 |
| FACIT-SP - GF | GF2 | 1 |  | 1 |
|  | GF4 | 2 |  | 2 |
|  | GF5 | 1 | 1 |  |
| FACIT-SP - SP12 M/P | SP2 | 1 | 1 |  |
|  | SP3 | 2 | 1 | 1 |
|  | SP4 | 2 | 1 | 1 |
|  | SP6 | 3 | 2 | 1 |
| FACIT-SP - SP12 F | SP9 | 1 |  | 1 |
|  | SP10 | 1 |  | 1 |
|  | SP11 | 2 | 1 | 1 |
|  | SP12 | 1 |  | 1 |
| SNAP Psychosocial needs | SNAP2 | 1 |  | 1 |
|  | SNAP3 | 2 | 2 |  |
|  | SNAP4 | 1 | 1 |  |
|  | SNAP7 | 1 |  | 1 |
| SNAP Spiritual needs | SNAP8 | 1 |  | 1 |
|  | SNAP13 | 1 | 1 |  |
|  | SNAP15 | 1 |  | 1 |
|  | SNAP16 | 2 | 2 |  |
|  | SNAP17 | 1 |  | 1 |
|  | SNAP18 | 1 | 1 |  |
| SNAP Religious needs | SNAP21 | 1 |  | 1 |
|  | SNAP23 | 1 | 1 |  |

**Supplemental Table 2** – Spiritual needs according to the patient's gender (mean and min–max).

|  | Total | Female (n=87) | Male (n=63) | p |
| --- | --- | --- | --- | --- |
| Psychosocial needs | 14.25 (5-20) | 14.67 (5-20) | 13.68 (7-19) | 0.042 |
| Spiritual needs | 34.72 (13-52) | 35.38 (13-52) | 33.81 (13-51) | 0.279 |
| Religious needs | 12.13 (5-20) | 12.31 (5-20) | 11.87 (5-20) | 0.541 |
| SNAP score | 61.09 (23-92) | 62.37 (23-92) | 59.32 (25-87) | 0.186 |

SNAP = Spiritual Needs Assessment for Patients.

**Supplemental Table 3** – Illustrative quotes.

| Category 1: Approach by the health care team | |
| --- | --- |
| Subcategory 1A:  The relevance of the topic and its pertinence for the team to approach. | “I believe religion and science should go hand in hand.” (P12)  “I believe spirituality and religiosity are important pillars of cancer treatment (…).” (P21)  “It would be helpful for doctors and staff to know a little about their patients' faith. This knowledge would allow them to better understand and help their patients regarding treatment.” (P140) |
| Subcategory 1B:  The means for this approach. | “There is no time during the follow-up treatment.” (P44)  “I don't think the team is willing to address the issue.” (P90)  “Conversations should come naturally. Sometimes we feel comfortable opening up, and sometimes we don't. However, these kinds of conversations must come naturally.” (P130) |
| Category 2: Comprehension of spirituality / religiosity. | |
| Subcategory 2A:  Religion and religiosity. | “As a believer, I have faith that Jesus Christ knows everything and cares about it.” (P81)  “I am a nonpracticing Catholic.” (P154) |
| Subcategory 2B:  Spirituality not linked to religion or religiosity. | " I believe that everyone tries to discuss the subject with their friends and family." (P90)  "I believe that everything makes sense, with positive energy and a kind of immersion into the true self." (P118) |
| Subcategory 2C:  The wish to learn more about spirituality / religiosity. | " Reflecting on the matter would help us." (P63)  " I don't see the need for that right now." (P162) |
| Category 3: Impact of the spirituality/religiosity. | |
| Subcategory 3A:  Potential benefits. | “I believe discussing this topic in a hospital setting can offer comfort, motivation, and support when dealing with the challenges of cancer treatment.” (P9)  “I like hearing that. It helps ease the anxiety that comes with treatment.” (P135) |
| Subcategory 3B:  Potential harms. | “I've been going through a difficult time with regard to my faith. I hope it passes soon because it's been difficult thus far.” (P35)  “Everyone has their own religion, and sometimes I may disagree with theirs.” (P42)  “Because it can lead to conflicts of opinion about religion.” (P164) |
| Category 4: Self-referential or other-referential responses. | |
| Subcategory 4A:  Other-referential. | “Learning about spirituality and discussing it with others is important. Hearing different perspectives can strengthen your faith and offer comfort during difficult times.” (P81) |
| Subcategory 4B:  Self-referential. | "Personally, I don't see the need for it. It's a very personal matter. I have my own faith." (P36) |
| Subcategory 4C:  Self-referential and other-referential. | "I am very reserved, but I believe it is important to other people." (P27)  " Personally, I don't see the need because I'm very clear about my beliefs. I'm not sure if others would be interested in discussing the topic. My beliefs are fundamental to my ability to experience this moment less painfully. However, I can't predict how people would react to discussing this topic in a hospital." (P152) |

P = participant.

**Supplemental Table 4** – Frequency of units according to the treatment intention: curative ( C ) or palliative ( P ) and wish for approach.

|  |  | Treatment intention | | Would you like the healthcare team to talk? | |
| --- | --- | --- | --- | --- | --- |
| Categories and subcategories | Total (n=123) | Curative  (n=75) | Palliative  (n=75) | No (n=46) | Yes (n=77) |
| I Approach by the health team | 94 | 52 | 42 | 31 | 63 |
| A The relevance of the topic and its pertinence for the team to approach | 36 | 19 | 17 | 10 | 26 |
| B The means for this approach | 58 | 33 | 25 | 21 | 37 |
| II Comprehension of spirituality / religiosity | 162 | 88 | 74 | 53 | 109 |
| A Religion and religiosity | 65 | 35 | 30 | 15 | 50 |
| B Spirituality not linked to religion / religiosity | 61 | 33 | 28 | 17 | 44 |
| C The wish to learn more about spirituality / religiosity | 36 | 20 | 16 | 21 | 15 |
| III Impact of the spirituality / religiosity approach | 87 | 36 | 51 | 12 | 75 |
| A Potential benefits | 76 | 28 | 48 | 3 | 73 |
| B Potential harms | 11 | 8 | 3 | 9 | 2 |
| IV Self-referential and other-referential responses | 123 | 61 | 62 | 46 | 77 |
| A Other-referential | 59 | 23 | 36 | 14 | 45 |
| B Self-referential | 55 | 33 | 22 | 27 | 28 |
| C Self-referential and other-referential | 9 | 5 | 4 | 5 | 4 |

**Supplemental Table 5 –** Pearson correlation coefficients between DUREL religiosity indexes and quality of life scores.

|  | GP | GS | GE | GF | FACIT-G | M/P | F | SP | FACIT-SP |
| --- | --- | --- | --- | --- | --- | --- | --- | --- | --- |
| OR | 0.094 | 0.046 | 0.062 | 0.089 | 0.099 | 0.216 | 0.461 | 0.371 | 0.203 |
| NOR | 0.127 | 0.064 | 0.017 | 0.007 | 0.076 | 0.186 | 0.538 | 0.385 | 0.191 |
| IR | 0.022 | 0.132 | 0.011 | -0.031 | 0.039 | 0.170 | 0.616 | 0.410 | 0.173 |

GP = physical well-being; GS = social and family well-being; GE = emotional well-being; GF = functional well-being; M/P = meaning and peace well-being; F = faith well-being; SP = spiritual well-being; OR = organizational religiosity; NOR = non-organizational religiosity; IR = intrinsic religiosity.
